# Obituary

**Published:** 1878-04

**Authors:** 


### Obituary.

### LUNDSFORD PITTS YANDELL, SR.

It becomes our sad duty to record the death of Dr. L. P. Yandell, Sr. Known as he was personally to many of our readers, and by reputation to all, they will receive the announcement sorrowfully.

Dr. Yandell died upon the morning of February 4th, after a short illness with pneumonia. He was born July 4th, 1803, and was consequently in his 75th year when he died. He was a native of Tennessee, born near Hartsville, Sumner county. He was the son of Dr. Wilson Yandell, a native of North Carolina, and an eminent practitioner in his day. Dr. Yandell attended his first course of lectures in the Transylvania University at Lexington, his second course in the University of Maryland at Baltimore, where he graduated in 1825. In 1831 he was called to the Chair of Chemistry in the Transylvania College, which position he held for six years, when (1837) he came to Louisville and assisted in the organization of the Medical Institute, which subsequently became the medical department of the University of Louisville. He filled at different times in this institution the chair of Chemistry, Materia Medica, and Physiology. Associated with him in the faculty of the University were Caldwell, Miller, Drake, Gross, Austin Flint, J. B. Flint, Cobb, Bartlett, and other celebrities in American Medicine. He continued in the University until 1858, when he removed to Memphis, Tenn., and for a year or so was professor of practice in a medical school which was attempted there. During the war he was for awhile in the hospital service of the Confederacy. In 1862 he was licensed to preach by the Memphis Presbytery, and for awhile was pastor of a church in Dyceton, Tenn. Returning to Louisville after the war, he has resided here ever since.—*Louisville Medical News*, February 9th, 1878.

---

### Dr. Edmund Randolph Peaslee.

Dr. Edmund Randolph Peaslee died on January 21st, at the age of sixty-three years, of pneumonia, contracted by exposure resulting from unusually pressing professional engagements. He was born in Newton, N. H., was educated at Dartmonth College and graduated in 1836. He graduated from Yale Medical College in 1840. During the following year he began the practice of his profession at Hanover, N. H., and also commenced the delivery of a series of lectures on anatomy and physiology at Dartmouth College. He became a professor of those two branches in 1842, and continued to hold that chair until the year 1870. In the year 1843 he was appointed lecturer on anatomy and surgery at Bowdoin College, and was professor of these branches of education during the period from 1845 to 1857, when he gave up anatomy, but continued to act as professor of surgery until 1860. Dr. Peaslee was appointed professor of physiology and general pathology, in the year 1851, at the New York Medical College, and from 1858 to 1860 he accepted the professorship of obstetrics in the same institution. He was elected professor of gynæcology at Dartmouth Medical College in 1872, and at Bellevue Hospital Medical College in 1874. He practiced seventeen years in Hanover, and subsequently in New York. He published a work on Human Histology, and also one one Ovariotomy. His distinguished services in his specialty and his skill as an ovariotomist are well known.—*Boston Medical and Surgical Journal.*

